# Enhancement of pooled chilled semen quality in four Kintamani dogs through extenders enriched with different concentrations of *Spondias pinnata* leaf extract

**DOI:** 10.5455/javar.2026.m1040

**Published:** 2026-06-22

**Authors:** Anak Agung Gde Oka Dharmayudha, I Ketut Puja, I Wayan Nico Fajar Gunawan, Wiwik Misaco Yuniarti, Bambang Sektiari Lukiswanto

**Affiliations:** 1 Doctoral Program Student, Faculty of Veterinary Medicine, Airlangga University, Surabaya 60115, Indonesia; 2 Laboratory of Veterinary Clinical Diagnosis, Clinical Pathology, and Radiology, Faculty of Veterinary Medicine, Udayana University, Denpasar 80234, Indonesia; 3 Laboratory of Veterinary Genetic and Reproductive Technology, Faculty of Veterinary Medicine, Udayana University, Denpasar 80234, Indonesia; 4 Department of Veterinary Clinic, Faculty of Veterinary Medicine, Airlangga University, Surabaya 60115, Indonesia

**Keywords:** Antioxidant, chilled semen, DNA integrity, motility, *Spondias pinnata*, viability

## Abstract

**Objectives:** The present study determined the effects of the addition of *Spondias pinnata* (L.f.) Kurz leaf extract to extender on the quality of Kintamani dog semen during storage at 5°C for 72 h.

**Materials and Methods:** Ejaculates were collected via manual stimulation from four dogs. The semen was pooled and diluted using a Tris-based extender, then divided into four treatment groups. Group T1: Control samples without extract; Group T2: Samples supplemented with *Spondias pinnata* leaf extract at 50 µg/100 ml; Group T3: Samples supplemented with 100 µg/100 ml; and Group T4: Samples supplemented with 150 µg/100 ml. The samples were evaluated for progressive motility, percent viability, and DNA integrity after storage for 0, 24, 48, and 72 h.

**Results:** The findings indicate that a coconut water-based Tris-citrate extender is a viable alternative for canine semen preservation. Supplementation with 50 µg/100 ml *S. pinnata* leaf extract provides measurable benefits for maintaining sperm quality during chilled storage. Additionally, the extender system was able to preserve DNA integrity for at least 72 h.

**Conclusions:** The use of *S. pinnata* leaf extract at an optimal concentration (50 µg/100 ml) improves progressive motility and viability of chilled Kintamani dog semen, while DNA integrity remains unaffected by storage duration or extender type. The coconut water-based Tris-citrate extender enriched with a moderate dose of *S. pinnata* leaf extract represents a promising formulation for short-term semen preservation. These findings may support the development of more accessible and cost-effective semen preservation techniques in Indonesia, particularly for local breeds such as the Kintamani dog.

## 1. Introduction

Artificial insemination (AI) has become an essential reproductive technology in canine breeding programs, particularly for the conservation, genetic improvement, and control of genetic diversity [1]. The Kintamani dog, an indigenous breed from Bali, Indonesia, possesses unique phenotypic and behavioral characteristics that have recently gained national and international recognition. The Kintamani dog is very famous among Indonesian people [2]. As interest in its conservation and selective breeding continues to increase, the development of efficient semen preservation techniques is crucial to support wider distribution of genetic material, minimize the need for animal transport [3], and enhance overall reproductive success [4].

One of the most commonly used methods to preserve canine semen is chilled storage. Keeping semen refrigerated for extended periods helps maintain its quality, allowing samples to be shipped over long distances. This approach eliminates the need to transport breeding males, thereby reducing stress, lowering transportation costs, and minimizing potential health risks associated with animal movement [5]. However, exposure to cold temperatures can lead to cold shock, oxidative stress, and structural deterioration of sperm cells [6]. These detrimental effects result in decreased progressive motility, reduced membrane integrity, lower fertility rates, and compromised DNA stability. To mitigate such damage, semen extenders are formulated to provide nutritional support and osmotic balance while incorporating antioxidants to neutralize reactive oxygen species (ROS) generated during storage [7].

In recent years, there has been growing interest in the application of natural plant-derived antioxidants in semen extenders due to their biocompatibility, availability, and potential protective effects [8], acting as antioxidants, preserving membrane and DNA integrity, and modulating apoptotic pathways [9]. Several plants, such as African basil leaf (*Ocimum gratissimum*), *Moringa oleifera*, and *Kaempferia parviflora*, have been demonstrated to have strong antioxidant capacity to maintain motility, viability, and DNA integrity [10, 11, 12]. *Spondias pinnata* (L.f.) Kurz is a medicinal plant traditionally used in various regions of Asia [13]. Phytochemical analyses have shown that this plant contains flavonoids, phenolic compounds, and other bioactive substances known for their strong antioxidant, anti-inflammatory, and cytoprotective properties [14]. These characteristics suggest that *S. pinnata* extract may offer significant protective benefits to spermatozoa exposed to oxidative stress during chilling. Despite its pharmacological potential, research investigating the use of *S. pinnata* extract in reproductive biotechnology, particularly in canine semen preservation, remains limited.

During storage, spermatozoa gradually experience a decline in motility and viability due to the accumulation of ROS [15]. Sperm cells are particularly susceptible to oxidative stress because their plasma membranes contain a high proportion of polyunsaturated fatty acids (PUFAs) [16]. Excessive ROS can induce lipid peroxidation of the sperm membrane, resulting in loss of membrane integrity and damage to cellular components such as proteins and DNA [17]. These alterations ultimately decrease sperm motility and viability during storage. The addition of antioxidants to semen extenders can help reduce these effects, as antioxidants function by neutralizing ROS and preventing the oxidative damage to sperm cells.

Considering the need to improve chilled semen quality and the potential advantages of natural antioxidants, this study aims to evaluate the effects of supplementing a Tris-based extender with *S. pinnata* leaf extract on the quality of chilled Kintamani dog semen. Semen quality parameters, including progressive motility, viability percentage, and DNA integrity, were assessed at multiple storage intervals. The findings of this research are expected to provide valuable insights into the application of plant-derived antioxidants in canine semen preservation and contribute to the enhancement of reproductive management strategies for the Kintamani dog breed.

## 2. Materials and Methods

### 2.1. Ethical approval

All procedures related to the handling and treatment of the animals during semen collection were approved by the Ethics Committee of the Faculty of Veterinary Medicine, Udayana University, Indonesia (Approval No. B/204/UN14.2.9/PT.01.04/2025).

### 2.2. Animal

The sample requirements for this research involve semen from home-kept Kintamani Bali dogs subjected to thorough evaluations of their breeding history, medical records, and medication or supplement intake for at least 6 months before collection. The donor dogs were healthy and between 2 and 3 years old. All procedures involving these dogs strictly adhered to the ethical guidelines of the Ethics Committee, Faculty of Veterinary Medicine, Udayana University, Indonesia. The inclusion and exclusion criteria are presented in Table 1. Semen samples were collected from four adult male Kintamani dogs aged 2–3 years and weighing 12.6–15.6 kg, which met all the inclusion criteria. All dogs were clinically healthy and had a body condition score of 5/9. They were fed a commercial diet twice daily and provided with unlimited access to water (*ad libitum*). The dogs have not been exposed to bitches in season.

**Table 1.** The inclusion and exclusion criteria of the Kintamani dogs.

No	Inclusion criteria	Exclusion criteria
1	Clinically healthy aged between 2 and 3 years	Clinically not healthy, and the age does not meet the requirement (not between 2 and 3 years)
2	Progressive motility ≥ 80% before treatment	Progressive motility < 80% before treatment
3	The ejaculate volume (0.5–2 ml)	The ejaculate volume is less than 0.3 ml or > 3 ml
4	There were sperm morphology abnormalities < 20% before treatment	The sperm morphology abnormalities were > 20% before treatment
5	Free contamination from urine, blood, or excessive debris	Contaminated with urine, blood, and/or excessive debris
6	The donor has been vaccinated and is free from infectious reproductive diseases	The donor has not been vaccinated or heavily stressed during collection

### 2.3. Semen collection

Ejaculates were collected using the manual stimulation method [18]. The second fraction was collected, which is sperm-rich; gathered into a plastic tube; and transported to the laboratory within a few min in an icebox maintained at 4°C. Immediately after collection, each sample was evaluated and had to meet the inclusion criteria (Table 1). After the evaluation, all semen samples were mixed together to avoid individual variability.

### 2.4. Spondias pinnata leaf extract

The extraction of active compounds from *S. pinnata* leaves was performed using a cold maceration method with distilled water to prevent the degradation of thermolabile compounds [19]. A total of 100 gm of dried *S. pinnata* leaf powder (simplisia) was placed into a dark-colored maceration vessel, followed by the addition of 1000 ml of distilled water (1:10 ratio). The maceration process was conducted for 3 × 24 h at room temperature, protected from direct sunlight, with periodic stirring every 24 h to maximize the diffusion of secondary metabolites [20].

Upon completion of maceration, the mixture was sequentially filtered through a flannel cloth and Whatman No. 1 filter paper to separate macroparticles from the filtrate. The resulting aqueous filtrate was then concentrated using a rotary evaporator at a maximum temperature of 50°C (or a freeze dryer) until the solvent was completely evaporated, yielding a thick aqueous extract of *S. pinnata* leaf [21]. The qualitative analysis of the extract was carried out using UV-Vis spectrophotometry at the Integrated Research Laboratory, Faculty of Mathematics and Natural Sciences, Udayana University, Indonesia. The *S. pinnata* leaf extract contains total phenolics 12.287 mg GAE/100 gm, total flavonoids 1.185 mg GAE/100 gm, total tannins 15.225 mg TAE/100 gm, antioxidant capacity 25.895 mg GAEAC/kg, total vitamin C 10.891 mg/100 gm, total vitamin A 731 mg/100 gm, and IC 50% 87.3 ppm.

### 2.5. Research design

This study used an experimental method with a completely randomized design. Collected semen from Kintamani dogs was divided into four aliquots, which, diluted in a coconut water-based Tris citrate extender, contain 2.4 gm Tris, 1.4 gm citric acid, 0.8 gm fructose, 0.06 gm penicillin G sodium salt, 0.1 gm streptomycin, 20% (v/v) young coconut water, and distilled water up to 100 ml with a 1:2 ratio (semen:extender) at ambient temperature. *S. pinnata* leaf extract was added to the semen at different concentrations (0, 50, 100, and 150 µg/100 ml) according to the experimental design, and the samples were stored at 5°C. Each treatment had five replications. Semen quality parameters, including spermatozoa progressive motility, viability, and morphology, were evaluated at 0, 24, 48, and 72 h of storage. The dose determination in microliters per 100 ml (μl/100 ml) in the extender medium was based on the method of liquid-phase plant extract supplementation commonly used in semen preservation protocols [22, 23]. Doses 0, 50 μl/100 ml, 100 μl/100 ml, and 150 μl/100 ml were selected as the experimental range to determine the balance point (osmolarity and pH) between phenolic antioxidant supplementation and potential toxicity that could affect the *in vitro* microenvironment of canine spermatozoa.

### 2.6. Evaluation of sperm

Sperm progressive motility was examined following the general guidelines described by Johnston et al. [24]. The assessment was carried out on a microscope (Olympus BX53, Olympus, Tokyo, Japan) stage maintained at 37°C. Approximately 200 sperm cells were observed under 100x magnification. The evaluation was performed manually by the same highly experienced observer to minimize subjective variation. During progressive motility evaluation, attention was given to distinguishing progressively motile spermatozoa from those that exhibited non-progressive or no movement, as described by Ajadi and Gazal [25].

Spermatozoa viability was evaluated using the Eosin-Nigrosin differential staining method. One drop of semen was homogeneously mixed with Eosin-Nigrosin stain on a microscope slide, then drawn into a thin smear and air-dried [26]. Two hundred sperm cells were examined at 400x magnification. Viable spermatozoa appeared unstained and transparent, as their intact membranes prevented dye penetration [27]. In contrast, non-viable spermatozoa absorbed the eosin and were identified by their red coloration.

The sperm DNA integrity was evaluated using the Acridine Orange (AO) staining. A thin semen smear was fixed using Carnoy’s solution (a mixture of methanol and glacial acetic acid) for 2 h, then air-dried. After that, it was stained with AO solution for 5 min in a dark room [28]. Observations must be carried out under a fluorescence microscope (Olympus U-RFL-T, Olympus, Japan) at 400x magnification. Based on its working principle, spermatozoa with intact chromatin will emit a green fluorescence. Meanwhile, spermatozoa with denatured or fragmented DNA will emit a yellow, orange, or red fluorescence. For each sample, 100 cells were counted and classified with intact and denatured DNA [28].

### 2.7. Data analysis

The collected data was analyzed using repeated measures multivariate analysis. The assumption of sphericity was tested using Mauchly’s test. When the assumption was violated, the Greenhouse-Geisser correction was applied. When significant differences among treatments were detected, post-hoc tests were performed using the Least Significant Difference (LSD) test using SPSS Version 27 for Windows (IBM Corp., Chicago, IL, USA) [29].

## 3. Results

The results of observations on the characteristics of fresh semen from Kintamani dogs after pooling were cloudy or milky in color. The average sperm progressive motility ranged from 89 to 94%, and viability ranged from 90 to 95%, which were considered high and within the normal physiological range for Kintamani dogs‘ semen, suggesting good initial semen quality. The average pH was 6 to 7, which was within the acceptable range, and DNA integrity was notably high, ranging from 97 to 99. The average concentration, percentage of progressive motility, and percentage of viability and intact DNA integrity are shown in Table 2. At 0 h of storage, no significant differences were observed among treatments (progressive motility, viability, and DNA integrity) (*p* > 0.05), indicating similar initial semen quality.

**Table 2.** Means and standard deviations of the characteristics of fresh semen obtained from Kintamani dogs (*n* = 4).

Semen characteristics	Mean ± SD	Range
Spermatozoa concentration (×10^6^)	683.75 ± 89.57	565–780
Motility (%)	91.50 ± 2.21	89–94
Viability (%)	93.00 ± 2.16	90–95
Intact DNA integrity	98.00 ± 0.81	97–99
pH	6.50 ± 0.57	6–7

Progressive motility of Kintamani dog spermatozoa decreased significantly during chilled storage from 0 to 72 h in all treatment groups (*p* < 0.01). During storage, progressive motility gradually declined across all experimental groups. The different concentrations of *S. pinnata* leaf extract significantly affected sperm motility (*p* < 0.01). Post-hoc LSD test showed that treatments supplemented with 100 and 150 µg/100 ml extract resulted in lower motility compared to the groups without extract supplementation and 50 µg/100 ml extract concentrations. At 72 h of storage, the highest mean motility value was observed in the treatment supplemented with 50 µg/100 ml extract, whereas the lowest value was observed in the 150 µg/100 ml treatment. However, these differences were not statistically significant (*p* > 0.05) (Table 3).

**Table 3.** Effect of *S. pinnata* extract supplementation on canine sperm progressive motility during short-term preservation at 5°C.

Extender	Duration Of Storage (h)			
	0	24	48	72
CW-SP0	87.00 ± 0.70^aA^	68.00 ± 0.70^aB^	54.40 ± 1.14^aC^	40.40 ± 0.54^aD^
CW-SP50	87.2 ± 0.83^aA^	67.60 ± 0.54^aB^	54.20 ± 1.09^aC^	40.80 ± 0.83^aD^
CW-SP100	87.60 ± 0.54^aA^	66.20 ± 0.83^bB^	52.00 ± 1.22^bC^	40.20 ± 1.48^aD^
CW-SP150	87.40 ± 0.54^aA^	64.20 ± 1.48^cB^	50.20 ± 1.48^cC^	39.00 ± 1.11^aD^

**Note:** CW-SP0: coconut water–based TRIS citrate extender without *Spondias pinnata* extract (0 µg/100 ml); CW-SP50, CW-SP100, and CW-SP150: Extenders were supplemented with 50, 100, and 150 µg/100 ml of *Spondias pinnata* extract, respectively. Data are presented as mean ± SD. Different lowercase superscripts ^(a–d)^ within the same column indicate significant differences among treatments at the same storage time (*p* < 0.05). Different uppercase superscripts ^(A–D)^ within the same row indicate significant differences among storage times within the same treatment (*p* < 0.05). Data are presented as mean ± standard deviation of five replications.

**Figure 1.**
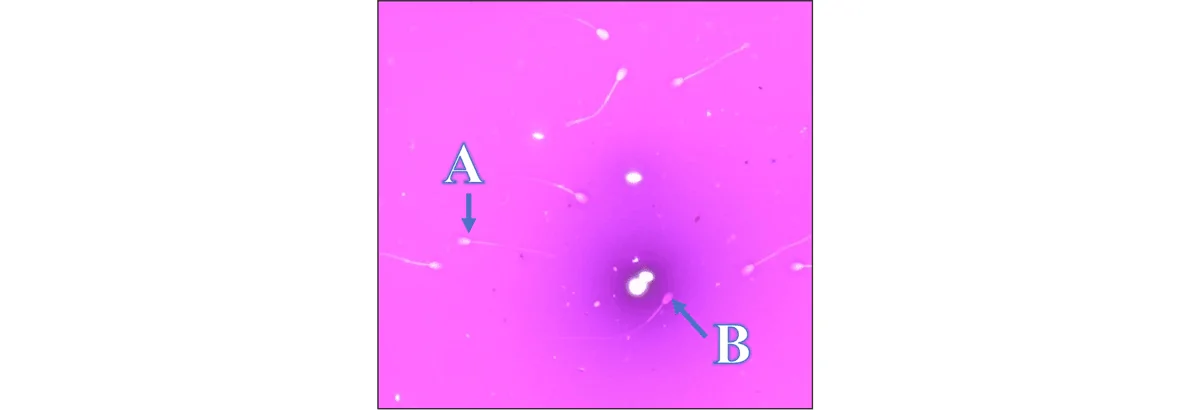
Viability assessment of spermatozoa shows live (unstained) spermatozoa (A) and dead (stained) spermatozoa (B).

Sperm viability decreased significantly during chilled storage from 0 to 72 h in all experimental groups (*p* < 0.01) (Figure 1). During storage, the concentration of *S. pinnata* leaf extract significantly affected sperm viability (*p* < 0.01). Based on the LSD post hoc test, the treatments containing 0 and 50 µg/100 ml extract differ significantly from each other (*p* < 0.05), and both showed significantly higher viability values compared with the treatments containing 100 and 150 µg/100 ml extract (*p* < 0.05). The lowest viability values were observed in the 150 µg/100 ml treatment, followed by the 100 µg/100 ml treatment. These results indicate that the concentration of *S. pinnata* leaf at 50 µg/100 ml is the best concentration to maintain sperm viability of Kintamani dogs during short-term preservation at chilled storage (Table 4).

**Table 4.** Effect of *S. pinnata* extract supplementation on canine sperm viability during short-term preservation at 5°C.

Extender	Duration Of Storage (h)			
	0	24	48	72
CW-SP0^a^	88.20±0.44^aA^	76.00±0.70^bB^	63.40±1.10^bC^	53.20±0.44^bD^
CW-SP50^b^	88.40±0.54^aA^	77.00±0.70^aB^	65.00±0.70^aC^	59.20±0.83^aD^
CW-SP100^b^	88.40±0.54^aA^	74.20±1.64^bB^	63.80±0.44^bC^	47.80±1.09^cD^
CW-SP150^c^	88.80±0.44^aA^	75.00±0.70^bB^	60.80±0.44^cC^	43.60±0.54^dD^

Note: CW-SP0: coconut water–based TRIS citrate extender without *Spondias pinnata* extract (0 µg/100 ml); CW-SP50, CW-SP100, and CW-SP150: Extenders were supplemented with 50, 100, and 150 µg/100 ml *Spondias pinnata* extract, respectively. Data are presented as mean ± SD. Different lowercase superscripts ^(a–d)^ within the same column indicate significant differences among treatments at the same storage time (*p* < 0.05). Different uppercase superscripts ^(A–D)^ within the same row indicate significant differences among storage times within the same treatment (*p* < 0.05). Data are presented as mean ± standard deviation of five replications.

**Figure 2.**
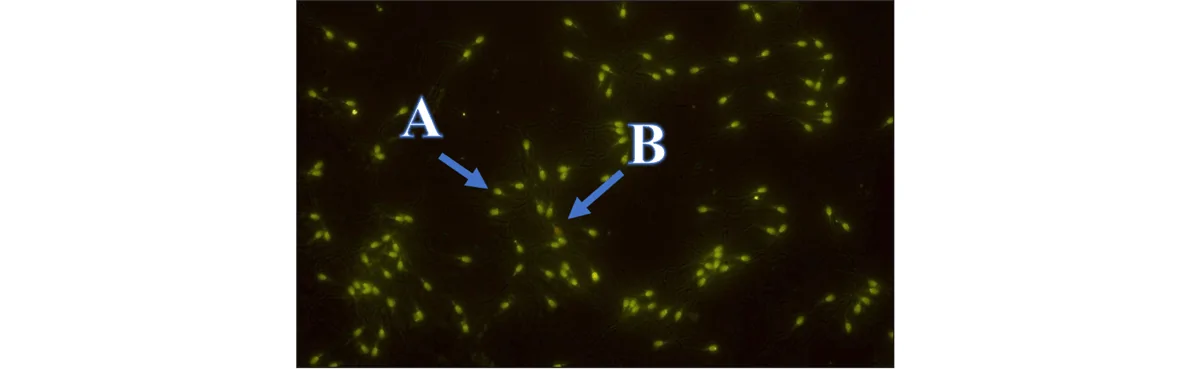
Representative fluorescent photomicrographs of Kintamani dog spermatozoa showing viable spermatozoa stained bright green (A) and spermatozoa with DNA fragmentation indicated by red fluorescence (B).

The statistical analysis showed that the supplementation of *S. pinnata* leaf extract had no significant effect on sperm DNA integrity during chilled storage (*p* > 0.05) (Figure 2). In addition, storage time did not significantly influence the percentage of spermatozoa with intact DNA (*p* > 0.05). The interaction between extract concentration and storage time was also not significant (*p* > 0.05). The values of DNA integrity remained relatively stable throughout the storage period in all treatment groups. These results indicate that the DNA integrity of Kintamani dog spermatozoa was consistently maintained during 72 h of chilled storage and was not affected by the addition of *S. pinnata* leaf extract at the tested concentrations (Table 5).

**Table 5.** Effect of *S. pinnata* extract supplementation on canine sperm DNA integrity during short-term preservation at 5°C.

Extender	Duration Of Storage (h)			
	0	24	48	72
CW-SP0^a^	98.00 ± 0.70^aA^	98.00 ± 0.00^aA^	98.00 ± 0.70^aA^	97.80 ± 0.44^aA^
CW-SP50^a^	98.40 ± 0.54^aA^	98.20 ± 0.44^aA^	98.00 ± 0.00^aA^	98.00 ± 0.00^aA^
CW-SP100^a^	98.20 ± 0.44^aA^	98.20 ± 0.44^aA^	98.20 ± 0.44^aA^	98.20 ± 0.44^aA^
CW-SP150^a^	98.40 ± 0.54^aA^	98.40 ± 0.54^aA^	98.20 ± 0.44^aA^	98.20 ± 0.44^aA^

Note: CW-SP0: coconut water–based TRIS citrate extender without *Spondias pinnata* extract (0 µg/100 ml); CW-SP50, CW-SP100, and CW-SP150: Extenders were supplemented with 50, 100, and 150 µg/100 ml of *Spondias pinnata* extract, respectively. Data are presented as mean ± SD. Different lowercase superscripts ^(a–d)^ within the same column indicate significant differences among treatments at the same storage time (*p* < 0.05). Different uppercase superscripts ^(A–D)^ within the same row indicate significant differences among storage times within the same treatment (*p* < 0.05). Data are presented as mean ± standard deviation of five replications.

## 4. Discussion

The present study investigated the effects of coconut water–based TRIS citrate extenders supplemented with *S*. *pinnata* leaf extract at varying concentrations on the motility, viability, and DNA integrity of chilled Kintamani dog spermatozoa stored at 5°C for up to 72 h. Overall, the findings demonstrate that the extender formulation and the concentration of *S. pinnata* extract influenced sperm quality parameters differently, with notable effects observed on motility and viability, while DNA integrity remained largely unchanged. These results contribute to the growing body of research on the use of plant-derived antioxidants to improve the longevity of chilled semen in domestic animals. Sperm need a fine balance between ROS and antioxidants. Too many oxidants cause oxidative stress, but too many antioxidants can cause reductive stress, both of which are damaging [30].

### 4.1. Quality of fresh semen and its importance in chilled storage

Fresh semen from Kintamani dogs exhibited excellent initial characteristics, including high motility (89–94%), high viability (90–95%), slightly acidic pH (6–7), and remarkably high DNA integrity (97–99%). These values are consistent with the expected physiological characteristics of healthy canine ejaculates and align with previous studies reporting similar ranges in dogs [2]. High-quality starting material is critical for semen preservation studies, as it ensures that observed changes are due to storage conditions and extenders rather than pre-existing deficits in semen quality.

The high intact DNA integrity in fresh semen also indicates minimal oxidative damage at the time of collection [31, 32]. Since spermatozoa are particularly prone to lipid peroxidation due to their high polyunsaturated fatty acid content [33], their initial status plays an essential role in determining their resilience during cold storage.

### 4.2. Effect on sperm progressive motility

The decrease in sperm progressive motility with increasing storage time observed across all extender groups is consistent with well-established effects of chilling injury. Cold temperatures reduce enzymatic activity, ATP production, and the functional capacity of mitochondria, all of which are essential for maintaining sperm motility. Furthermore, extended exposure to low temperatures increases the risk of oxidative stress and structural damage to the flagellar membrane, contributing to the progressive decline in motility [17, 33].

Despite this overall decline, the extenders supplemented with *S. pinnata* extract had differing effects on motility. Notably, the extender containing 50 µg/100 ml extract consistently produced the highest motility values over 72 h, although it is not statistically significantly different from extenders without *S. pinnata* leaf extract. This suggests that a moderate concentration of *S. pinnata* extract may enhance membrane stability or reduce oxidative stress to maintain motility during cooling.

The ineffectiveness of the highest concentrations (100 and 150 µg/100 ml) in maintaining motility and even its association with the lowest motility values may indicate a potential threshold beyond which antioxidant compounds begin to exert pro-oxidant effects or cause osmotic imbalance [34]. Such biphasic responses have been reported in the use of other plant extracts, such as green tea polyphenols, rosemary extract, and vitamin C, where high concentrations can destabilize membranes or impair mitochondrial function [35].

These results support the hypothesis that *S. pinnata* extract provides antioxidant protection that is dose-dependent and most effective at moderate concentrations. The plant is known to contain flavonoids, tannins, and phenolic acids, all of which have been documented to scavenge free radicals and inhibit lipid peroxidation mechanisms likely contributing to improved motility preservation [14].

### 4.3. Effect on sperm viability

The percentage of viability also showed a significant decline over time in all treatments, consistent with the natural deterioration of sperm cell membranes during chilled storage. The observed decline is likely associated with structural membrane damage, reduced membrane fluidity, and increased oxidative stress. These factors can compromise membrane integrity and result in increased numbers of dead sperm cells over prolonged storage.

Similar to the motility results, the extender containing 50 µg/100 ml *S. pinnata* extract produced the highest viability after 72 h and was statistically significant compared to the extender without extract supplementation and with 100 and 150 µg/100 ml concentrations. This emphasizes the potential role of *S. pinnata* extract as an effective membrane protector and antioxidant. Phenolic compounds may interact with sperm membranes to stabilize phospholipid bilayers and inhibit ROS accumulation, thus reducing apoptotic and necrotic pathways [36]. Phenolics have one or more aromatic rings with phenolic –OH groups. These donate a hydrogen atom or electron to radicals, converting them to less reactive species while forming a relatively stable phenoxyl radical [37]. Sperm plasma membranes are rich in PUFAs and highly vulnerable to lipid chain peroxidation, which impairs fluidity, ion channels, and acrosome reactions [38]. Phenolics can interrupt lipid peroxidation chains by trapping lipid peroxyl radicals, lowering MDA and 4-HNE, and preserving membrane fluidity and mitochondrial potential.

In contrast, the 150 µg/100 ml concentration yielded the lowest viability among treatment groups. This supports the notion that excessive antioxidant concentrations may disturb membrane osmolarity or increase oxidative activity through redox cycling, ultimately damaging rather than protecting sperm cells. Overuse of antioxidants may lead to reductive stress, which is reported to be as dangerous to cells as oxidative stress [39]. Taken together, these results may indicate that low to moderate concentrations of *S. pinnata* extract improve sperm survival during chill storage, whereas high concentrations may have negative effects.

### 4.4. Intact DNA integrity as a stable parameter during chilled storage

One of the most intriguing findings in this study is that DNA integrity remained remarkably stable across all extender types and storage durations. Unlike motility and viability, DNA integrity did not exhibit significant deterioration. This suggests that the conditions used in this study, including the cooling rate, the extender formulation, and the storage temperature, were adequate to prevent DNA fragmentation during the 72-h period.

Several studies have reported that mitochondrial dysfunction and membrane damage occur earlier than nuclear DNA damage during chilling [40, 41]. DNA is generally more resilient to cold-induced stress than plasma membranes [42], provided that oxidative stress levels are not excessively high. Thus, the preservation of DNA integrity observed in the present study supports the idea that the extenders used, including those supplemented with *S. pinnata* extract, were effective in minimizing oxidative damage.

Moreover, the consistently high DNA integrity values (> 97%) across all treatments suggest that Kintamani dog spermatozoa possess robust DNA structural stability under chilled conditions, further corroborating findings in canine reproductive studies that DNA integrity remains intact during short-term storage.

The ability to preserve motility and viability of dog semen for up to 72 h has important practical applications, including facilitating sperm transport between distant locations and improving the success of artificial insemination, particularly in regions where liquid semen storage is preferred to cryopreservation.

The effectiveness of *S. pinnata* leaf extract in maintaining spermatozoa quality during storage periods longer than 72 h still needs to be further evaluated. Prolonged semen storage may increase the accumulation of ROS, which originates from the residual metabolism of spermatozoa as well as from cellular damage during cold storage. Therefore, further studies are required to evaluate the effectiveness of *S. pinnata* extract in maintaining semen quality during longer storage durations.

In addition to cold storage, cryopreservation, or semen freezing may produce different responses to antioxidant treatments. The freezing and thawing process is known to cause structural and functional damage to spermatozoa due to osmotic pressure changes, ice crystal formation, and increased oxidative stress. These damages may include disruption of plasma membrane integrity, mitochondrial dysfunction, and increased sperm DNA fragmentation, which ultimately reduce fertilization capability [43, 44]. Therefore, further research is needed to evaluate whether the addition of *S. pinnata* leaf extract in a tris–coconut water extender can also provide protective effects on spermatozoa during the cryopreservation process.

## 5. Conclusions

In summary, the use of *Spondias* *pinnata* extract at an optimal concentration (50 µg/100 ml) improves motility and viability of chilled Kintamani dog semen, while DNA integrity remains unaffected by either storage time or extender type. The coconut water-based Tris citrate extender supplemented with moderate doses of *S. pinnata* extract thus represents a promising formulation for short-term semen preservation. Future studies should investigate fertility outcomes following insemination and explore synergistic combinations of plant-derived antioxidants.

## Data Availability

The data presented in this study is available from the corresponding author upon reasonable request.

## List of abbreviations

4-HNE, 4-hydroxynonenal; AI, artificial insemination; AO, acridine orange; ATP, adenosine triphosphate; DNA, deoxyribonucleic acid; GAE, Gallic Acid equivalent; GAEAC, Gallic Acid equivalent antioxidant capacity; LSD, least significant difference; MDA, malondialdehyde; ROS, reactive oxygen species.
